# Factors associated with women’s healthcare decision-making during and after pregnancy in urban slums in Mumbai, India: a cross-sectional analysis

**DOI:** 10.1186/s12889-022-13216-7

**Published:** 2022-04-13

**Authors:** Neha Batura, Stavros Poupakis, Sushmita Das, Ujwala Bapat, Glyn Alcock, Jolene Skordis, Hassan Haghparast-Bidgoli, Shanti Pantvaidya, David Osrin

**Affiliations:** 1 Institute for Global Health, University College London, 30 Guilford Street, London, WC1N 1EH UK; 2 Society for Nutrition, Education and Health Action, Mumbai, India; 3 UCL Centre for the Health of Women, Children and Adolescents, London, UK

**Keywords:** Agency, Healthcare utilisation, Maternal health, India

## Abstract

**Background:**

Understanding factors associated with women's healthcare decision-making during and after pregnancy is important. While there is considerable evidence related to general determinants of women’s decision-making abilities or agency, there is little evidence on factors associated with women's decision-making abilities or agency with regards to health care (henceforth, health agency), especially for antenatal and postnatal care. We assessed women’s health agency during and after pregnancy in slums in Mumbai, India, and examined factors associated with increased participation in healthcare decisions.

**Methods:**

Cross-sectional data were collected from 2,630 women who gave birth and lived in 48 slums in Mumbai. A health agency module was developed to assess participation in healthcare decision-making during and after pregnancy. Linear regression analysis was used to examine factors associated with increased health agency.

**Results:**

Around two-thirds of women made decisions about perinatal care by themselves or jointly with their husband, leaving about one-third outside the decision-making process. Participation increased with age, secondary and higher education, and paid employment, but decreased with age at marriage and household size. The strongest associations were with age and household size, each accounting for about a 0.2 standard deviation difference in health agency score for each one standard deviation change (although in different directions). Similar differences were observed for those in paid employment compared to those who were not, and for those with higher education compared to those with no schooling.

**Conclusion:**

Exclusion of women from maternal healthcare decision-making threatens the effectiveness of health interventions. Factors such as age, employment, education, and household size need to be considered when designing health interventions targeting new mothers living in challenging conditions, such as urban slums in low- and middle-income countries.

## Background

In low- and middle-income countries (LMICs), including countries in South Asia, women have less agency i.e. the ability to make choices, than men [1, 2], and than women in resource-rich settings [3]. Several studies have shown that the exclusion of women from decision-making processes, and women’s lack of control over financial and non-financial resources, can lead to adverse effects on family well-being and development [4]. These include poor maternal health outcomes [5, 6], poor nutritional outcomes of children, [7, 8], and chronic household poverty [4]. Women’s agency is therefore closely linked with maternal and child health outcomes [9], and is crucial to the effective implementation of health programmes, especially in LMICs [10].

Substantial evidence describes the demographic, social and economic factors associated with women’s agency [11–16], and the association between women’s agency and maternal and child health outcomes [7, 17, 18]. However, there is less evidence on factors associated with women's decision-making abilities or agency with regards to perinatal health care, especially in economically deprived areas. This study aimed to fill this gap by assessing women’s agency in perinatal healthcare decision-making (henceforth, health agency) and examining factors associated with increased participation in healthcare decisions in slums in Mumbai, India.

## Methods

### Setting

Mumbai, the capital city of Maharashtra, has a population of 13.1 million residents and is characterised by wide socio-economic disparities [19]. Over half (55%) of the city’s residents live in slums [20, 21]. Despite this disparity in economic status, indicators of women’s agency such as literacy, work, workforce participation, age at marriage, and healthcare seeking tend to be higher than the national averages [22].

Our study uses data collected between 2006 and 2009 during a cluster randomized controlled trial of community mobilization to improve maternal and newborn health [23, 24]. The sampling frame for the trial was six municipal wards. From each of these wards, eight slum clusters in the catchment areas of 24 previously identified health posts were randomly selected (48 clusters in total). Social workers external to the trial drew lots to select 48 in blocks of eight per ward, and then to allocate four clusters per block to the intervention and control arms. Each cluster comprised approximately 1,000 households and covered a population of approximately 283,000 [23, 24]. The cluster separation was wide enough to minimize contamination (for further details, please refer to the trial protocol [24]). There were no statistically significant differences in maternal and newborn health outcomes between the intervention and control clusters [23]. As the dwellings were ‘informal structures’, 26% of homes were of insubstantial fabric (corrugated iron, planking, tarpaulin). Access to basic amenities was on average limited compared to other urban areas in India. For example, 28% of households in our clusters did not have metered electricity, compared to 7% in urban areas in India overall. Most households in our clusters had access to shared public toilets, while 59% of households in urban India had access to individual toilets [25, 26]. However, only 21% of households in the clusters did not have access to individual or communal piped water, compared to 29% of households in urban areas in India. More than one-third of clusters were adjacent to environmental hazards such as open garbage disposal areas, polluted bodies of water and railway lines [26].

### Ethical approval

This study is part of a cluster-randomised controlled trial of community mobilisation in Mumbai slums to improve care during pregnancy, delivery, postpartum and for the newborn. Data for this study was concurrent to the data collection process of the trial, which was part of the City Initiative for Newborn Health approved by the Municipal Corporation of Greater Mumbai and the Independent Ethics Committee for Research on Human Subjects, Mumbai, India (ref: IEC/06/31). All data collection and analytical methods were carried out in accordance with relevant guidelines and regulations. Verbal informed consent was sought from all participants for participation and dissemination of findings.

### Data collection

In each community, live births, stillbirths, maternal and neonatal deaths were recorded using a community surveillance system involving 99 locally-resident women. A trained investigator interviewed mothers 6 weeks after giving birth and asked questions on demographic characteristics, socioeconomic indicators, and maternal and newborn care. A questionnaire that included questions on health agency was administered to a sub-sample of women living in the study area who gave birth from March to September 2009 and consented to participate. The questions were adapted from Demographic and Health Survey questions [22] and a review of the literature on methods for identifying and measuring women’s agency and empowerment [27–31].

Owing to the restricted availability of young mothers and the sensitive nature of the questions, most of the interviews were conducted in participants’ homes and researchers made efforts to interview women alone. However, the density of slum homes and a desire to make respondents comfortable posed a challenge to achieving complete privacy. Participants decided whether there was sufficient privacy to commence the interview or reschedule. If family members were present, the researcher explained to the family that it might be embarrassing for the participant to answer some questions in their presence. Most family members agreed to privacy and often left before the agency questionnaire was administered.

In the absence of reliable estimates for agency, the sample size was based on estimates used for an intimate partner violence study which was also part of the cluster-randomised controlled trial described above [26]. Given available estimates of the prevalence of intimate partner violence, a sample size of 1,800 would give an estimated proportion with a precision of 5% at a confidence level of 95%. Sequential recruitment was planned until 300 questionnaires had been collected in each of the six municipal wards across the 48 communities. Because of the sensitive nature of the questions, we anticipated a high attrition rate, leading to a larger sample size of 2,630 participants.

### Variables

The health agency module included nine questions on women’s participation in decisions about (1) going for antenatal care in the first three months of pregnancy, (2) increased rest during pregnancy, (3) increased intake of food during pregnancy, (4) where to deliver the baby, (5) giving the baby colostrum, (6) care-seeking for herself, (7) care-seeking for her baby within the first month after birth, (8) feeding other than breastmilk, and (9) the baby’s immunisation. The responses to these questions were categorical: the woman decided by herself; the woman and her husband made a joint decision; the woman gave her opinion; and the woman’s husband and family made the decision. The category ‘woman gave her opinion’ was excluded from the analysis as it had a frequency of less than 1%. For each respondent, we created an index for health agency based on these nine variables (giving ‘woman decided by herself’ and ‘woman and her husband made a joint decision’ the same score) using principal components analysis [36]. We combined all nine variables into one index rather than creating separate indices for health agency in the antenatal and postnatal periods for two reasons: a) we are interested in health agency over the perinatal period; and b) to create a more robust index. The first principal component accounted for 80% of the variability and was used as an indicator of women’s participation in decisions about health care-seeking during pregnancy and for her newborn.

Data were also collected on women’s age at interview, age at marriage, level of education, religion, engagement in paid employment in the last 12 months, size of the household, and a set of questions used to create a composite asset score as an indicator of socioeconomic status of the household (assets included mattress, pressure cooker, gas cylinder, stove, chair, cot, table, clock, fan, cycle, radio, sewing machine, phone, fridge tv, motorbike and car) [32, 33].

### Data analysis

We used descriptive analysis to describe the health agency variables and the factors examined in this study. The health agency variables were expressed as percentages. For the health agency score and each factor, we calculated the mean, standard deviation, 5^th^ percentile and 95^th^ percentile. Factors were chosen based on data availability, evidence from the literature, and the presence of a statistically significant association (*p*-value 0.05) with the health agency score using an unadjusted linear regression analysis.

We used multiple linear regression analysis to test the associations between health agency score and the decision factors in the final adjusted model. The final model included women’s age (in years), age at marriage (in years), household size (number of people), a categorical variable denoting household composition (nuclear or joint/extended), household asset score, employment (a binary indicator for being engaged in paid work in the last 12 months), and a categorical variable for education (using no education as a reference category and dummy variables for primary, secondary, and higher education). The final model included dummy variables for each of the 48 urban slum communities included in the sample to account for local community-level heterogeneity, such as access to public sanitation or health facilities. Standard errors were adjusted accordingly to account for intra-community correlation. All analyses were conducted using Stata 16.0 (Stata Corp, College Station, TX, USA).

## Results

Agency data were collected from 2630 women. Of these, 491 (18.6%) interviews were not completed as women declined to answer all the agency questions. A further 73 (3%) interviews were not completed because of lack of privacy. After cleaning the data, complete information was available for 2,017 women (77%) and we used these data in the analysis.

Tables 1 and 2 present an overview of socio-demographic profile of the women in our sample, and the extent of women’s participation in decisions about their health and their babies’ health. Overall, most women participated in decision-making, either making decisions themselves or jointly with their husbands. For example, a higher proportion of women reported making a decision themselves about taking more rest during pregnancy (52.8%), eating more during pregnancy (55.1%), giving the baby colostrum (51.1%), feeding the baby things other than breastmilk (45.6%), and infant immunization (44.0%), than women who reported making these decisions jointly with their husbands, or women who reported not participating in these decisions.

**Table 1:** Women's participation in healthcare decision-making during and after pregnancy (*N* = 2017)

	Participation in decision-making (%)		
Decision about	Woman alone	Woman & husband jointly	Husband & other family members
Antenatal care in first 3 m of pregnancy	34.8	30.1	35.1
More rest during pregnancy than before	52.8	20.9	26.3
Eating more during pregnancy than before	55.1	20.6	24.3
Place of delivery	29.6	33.7	36.7
Giving the baby colostrum	51.1	20.9	28.0
Seeking care for herself when ill	33.1	34.1	32.8
Seeking care for her baby when ill	31.8	35.5	32.6
Feeding her baby other than breastmilk	45.6	22.9	31.6
Infant immunisation	44.0	25.8	30.2

**Table 2:** Descriptive statistics of health agency score and factors examined (*N* = 2017)

Variable	Mean	Std. Dev	5^th^ Pctl	95^th^ Pctl
Health agency score	0.00	2.74	-4.52	2.00
Age (years)	24.54	4.16	19.00	32.00
Age at marriage (years)	19.04	3.07	14.00	25.00
Household size	6.31	3.07	3.00	12.00
Household asset score	0.15	0.94	-1.34	1.61
Engaged in paid work (last 12 months)	0.13	0.34	0.00	1.00
No education	0.21	0.41	0.00	1.00
Primary education	0.05	0.22	0.00	1.00
Secondary education	0.63	0.48	0.00	1.00
Higher education	0.11	0.31	0.00	1.00
Hindu	0.48	0.50	0.00	1.00
Muslim	0.46	0.50	0.00	1.00
Other religions (Buddhist, Christian, Sikh, etc.)	0.06	0.25	0.00	1.00
Nuclear family	0.46	0.50	0.00	1.00

However, fewer women reported making independent decisions about having antenatal care in the first 3 months of pregnancy (34.8%), the place of their delivery (29.6%) and seeking care for themselves when ill (33.1%) or for their baby when ill (31.8%) than women who reported making the decision jointly with their husbands or those who did not participate in the decision-making process.

These nine decision-making questions were used in the principal component analysis to create the health agency score of mean zero and standard deviation (SD) 2.74. Table 2 presents the mean and SD of this score and each variable examined as a factor. In addition, the 5^th^ and 95^th^ percentiles are reported as further measures of dispersion for all variables. The mean age at interview was 24.5 (SD 4.2) and age at marriage was 19.0 (3.1). The average household had 6.3 members (SD 3.1) and an asset score of 0.15 (0.9). Only a small fraction of the women in the sample had engaged in paid work in the last 12 months (13%). Most women had secondary education (63%), while fewer had none (21%), and even fewer had higher (11%) or primary (5%) education. Most women in the sample were Hindu (48%) or Muslim (46%). About half lived in a nuclear family (46%).

Table 3 shows the results of the linear regression analysis. The adjusted model revealed statistically significant associations with all the factors examined, except for household asset score (β -0.010, SE 0.084, p 0.903) and the main religion groups (β 0.288, SE 0.198, p 0.152;). However, there was a significant difference for those of other religions (β 0.373, SE 0.217, p 0.093) which we consider to be of limited interest as it represents only a small portion of the sample. There was a positive association between health agency score and age (β 0.121, SE 0.018, *p* < 0.001) which was also of large magnitude, as one SD increase in age was associated with a 0.2 SD increase in health agency score. An association of similar magnitude, but of opposite sign, was observed for household size (β -0.112, SE 0.030, p 0.001). Age at marriage was negatively associated with heath agency score (β -0.055, SE 0.024, p 0.025), although the association was of much smaller magnitude. In addition, age and age at marriage were, as expected, correlated (correlation co-efficient 0.22). Women who were engaged in paid work in the last 12 months had, on average, about half score unit (0.2 SD) higher (β 0.472, SE 0.183, p 0.013) than women who did not engage in paid work. There was an association with education, with women who had higher education having, on average, almost two-thirds of a score higher (β 0.560, SE 0.191, p 0.005) than women with no education. Finally, those in a nuclear family had, on average, a score one unit higher (0.4 SD, β 0.972, SE 0.180, *p* < 0.001) than those in a joint/extended family.

**Table 3:** Factors associated with health agency during and after pregnancy (*N* = 2017)

	**Unadjusted Model**			**Adjusted Model**		
**Factors**	β coef	Std. Error	*p*-value	β coef	Std. Error	*p*-value
Age (years)	0.148	0.013	< 0.001	0.121	0.018	< 0.001
Age at marriage (years)	-0.024	0.019	0.211	-0.055	0.024	0.025
Engaged in paid work (last 12 m)	0.610	0.162	< 0.001	0.472	0.183	0.013
Household size	-0.199	0.023	< 0.001	-0.112	0.030	0.001
Household asset score	-0.408	0.064	< 0.001	-0.010	0.084	0.903
No education		Ref			Ref	
Primary education	-0.218	0.295	0.459	0.227	0.291	0.439
Secondary education	-0.340	0.153	0.026	0.292	0.167	0.088
Higher education	-0.083	0.220	0.705	0.560	0.191	0.005
Hindu		Ref			Ref	
Muslim	0.199	0.126	0.116	0.288	0.198	0.152
Other religions	0.352	0.253	0.165	0.373	0.217	0.093
Nuclear family	1.642	0.115	< 0.001	0.972	0.180	< 0.001

## Discussion

This study aimed to explore the influence of key socioeconomic and demographic factors on women’s agency with respect to seeking care for themselves and their babies during and after pregnancy, in slums in Mumbai, India. Women are unlikely to exercise agency in choice of healthcare if they are not aware of the need and availability. In this sense, their potential agency is reduced before the opportunity to exercise it occurs. We cannot be sure that this was not the case for some respondents. However, it serves to underline the structural basis of lack of decision-making opportunity, in which awareness is limited and women are disempowered before the fact.

The regression results indicate that health agency increases with maternal age. This result is similar to other LMIC studies (Nepal, Bangladesh, India, Pakistan and Kenya), which find that women’s age is associated with increased agency, autonomy and decision-making power [9, 34–37].

Women who were educated and employed in the last 12 months had greater health agency. Studies from Nepal, Bangladesh, India and Pakistan [9, 34, 35, 37] also report similar findings. Women who have completed higher levels of education are more likely to be more physically and socially mobile, which increases opportunities to acquire, assimilate and engage with new information and knowledge [38]. These women are also more likely to contribute to their household’s income (directly or indirectly). This is considered to be a source of increased decision-making power relative to that of men [39] as it may increase a woman’s perceived contribution to her household’s economic status, and gives women a sense of economic independence, especially if they are employed outside their household. This would, of course, still depend on the decision-making mechanisms in the household [8]. Women who work outside their homes not only have enhanced capabilities, but also have more social contact with people other than household members [8]. Wider social contact can promote a clearer perception of well-being and individuality, which may enhance agency [38].

Greater health agency was also positively associated with living in smaller households and in nuclear families, but not in less wealthy households. These results are less straightforward to interpret. In urban settlements, living in large households is indicative of poverty: large households allocate an already small pool of resources among several people, where each individual would have a smaller share. This could be a barrier to health agency for women. A study in Pakistan reported similar findings [37]. However, an alternative view is that larger households are more established and more traditional than smaller or nuclear ones. In this case, household size might act as a proxy for religion and culture, creating strong familial ties that make it less easy for women to pursue individual courses of actions, as found in a study of women’s agency in Egypt [40]. In contrast, the absence of in-laws in nuclear families may allow women more opportunity to participate in decision-making [41]. Alternatively, smaller households or nuclear families could be more financially vulnerable or unstable, and women in these households are more likely to exhibit higher levels of agency in certain decisions because of absent husbands (or husbands with high-risk behaviours such as alcoholism and gambling) [42].

Our findings indicate that younger women, unemployed women and those with less education are likely to have lower health agency. Interventions targeted at women’s health should include these factors and incorporate supportive mechanisms and pathways to increase health agency (and agency overall). These may include improved access to economic and social resources in women’s families, they communities that they live in as well as markets for education and employment [43]. Having access to such opportunities could provide women with the potential to define their goals, enable them to act on them, achieving positive behaviour change and potentially improving their health (and other well-being) outcomes.

There are a few limitations to our study. First, it included women living in slums and cannot be generalised for the broader urban population or for rural areas. Second, the sensitive nature of the questions was likely to lead to social desirability bias. Some women might feel intimidated and misreport out of fear, especially when family members were close by during the administration of the health agency module. Third, other potential factors not identified in our study might confound the results, such as other family characteristics related to wealth, culture, or family composition. Fourth, the analyses are based on data from 2009. Survey data from the National Family Health Surveys from 2005–06 and 2015–16 show that in India, there are very small increases in the proportion of women included in household-level decision making, especially with regards to their own health [25, 44]. This indicates that levels of health agency have remained relatively stable over the last decade, and that these results are still relevant and could be helpful in the design of interventions to improve maternal health, especially those that focus on modifying behaviour of pregnant women to achieve optimal health outcomes.

## Conclusion

This analysis used cross-sectional data to explore factors associated with women’s health agency, created by a set of questions relating to participation in decision-making during and after pregnancy. Higher health agency is associated with age, employment, education and small household size. Health interventions targeted at women should incorporate mechanisms that can improve women’s agency; for example, opportunities for education, training and stable employment that could increase income earning potential and decision-making power within households; and expansion of social networks that create an experience of mutual support from individuals within and outside the household. These factors must be borne in mind when designing health interventions targeted at women in deprived, and resource poor settings.

## Data Availability

The datasets generated and/or analysed during the current study may be available from the corresponding author, and study team on reasonable request.

## Abbreviations

LMIC: Low- and middle-income country
SD: Standard deviation
SE: Standard error
